# Highly Water-Dispersible Graphene Nanosheets From Electrochemical Exfoliation of Graphite

**DOI:** 10.3389/fchem.2021.699231

**Published:** 2021-07-21

**Authors:** Si-Woo Park, Byungkwon Jang, Han Kim, Jimin Lee, Ji Young Park, Sung-Oong Kang, Yong-Ho Choa

**Affiliations:** ^1^ Department of Materials Science & Chemical Engineering, Hanyang University, Ansan, South Korea; ^2^ MExplorer Co., Ltd., Ansan, South Korea

**Keywords:** graphene, water dispersion, sulfate functionalization, electrochemical exfoliation, edge functionalization

## Abstract

The electrochemical exfoliation of graphite has been considered to be an effective approach for the mass production of high-quality graphene due to its easy, simple, and eco-friendly synthetic features. However, water dispersion of graphene produced in the electrochemical exfoliation method has also been a challenging issue because of the hydrophobic properties of the resulting graphene. In this study, we report the electrochemical exfoliation method of producing water-dispersible graphene that importantly contains the relatively low oxygen content of <10% without any assistant dispersing agents. Through the mild *in situ* sulfate functionalization of graphite under alkaline electrochemical conditions using a pH buffer, the highly water-dispersible graphene could be produced without any additional separation processes of sedimentation and/or centrifugation. We found the resulting graphene sheets to have high crystalline basal planes, lateral sizes of several μm, and a thickness of <5 nm. Furthermore, the high aqueous dispersion stability of as-prepared graphene could be demonstrated using a multi-light scattering technique, showing very little change in the optical transmittance and the terbiscan stability index over time.

## Introduction

Graphene, atomically thin carbon layers exfoliated from graphite in which multiple layers of sp^2^-bonded carbon atoms are arranged in a hexagonal lattice, has been standing at the center of materials due to its great potential applications in next-generation electronic devices (Kwon et al., 2020; Alonso et al., 2018), energy conversion and storage devices (Jang et al., 2021; Tanguy et al., 2020), catalysis (Hwang et al., 2019; Lu et al., 2016), and other functional composites (Ryu et al., 2020; Wu et al., 2017) owing to its unique electrical, mechanical, optical, and chemical properties (Kwon et al., 2017; Dong et al., 2017; Kim et al., 2019). While the preparation methods of graphene such as Scotch Tape exfoliation (Novoselov et al., 2004), epitaxial growth (Yang et al., 2013), and chemical vapor deposition using gaseous precursors (Plutnar et al., 2018) could yield high-quality graphene, its commercialization has been hindered owing to the lack of cost-effective industrial-scale production methods.

Among the alternatives, wet chemical approaches that operate *via* chemical reduction of graphene oxide (GO) and mechanical liquid-phase exfoliation of graphite such as ultrasonication or shear mixing have opened pathways to the mass production of graphene (Chuaa et al., 2014; Paton et al., 2014). In particular, the classical and well-known wet chemical route, oxidation of graphite into GO and its concurrent reduction of GO to reduced GO (rGO), has suffered from generating a large amount of hazardous wastes and poisonous gases while leaving inevitable structural defects within the as-reduced GO (Chuaa et al., 2014). In addition, the liquid-phase mechanical exfoliation of graphite into graphene has also presented the limits of small size and low yield of thin graphene nanosheets with respect to the use of solvent- or surfactant-assisted exfoliation using expanded graphite or graphite intercalation compounds (GICs) (Lee et al., 2009; Paton et al., 2014).

Recently, the electrochemical exfoliation of graphite has drawn attention as an easy, simple, and eco-friendly method to produce high-quality graphene (Parvez et al., 2014). When a voltage is applied to a graphite electrode, few-layer graphene sheets are exfoliated from the graphite layers over successive reaction steps, including the formation of GICs, expansion of the graphite layer through gas-evolving electrolysis, and exfoliation of graphite layers in various electrolytes (Parvez et al., 2014). The electrochemical exfoliation of graphite has been performed by changing the electrolyte type to control the characteristics of the resulting graphene sheets, such as their lateral size, thickness, and electronic and chemical properties (Yang et al., 2016). For the easy applications of graphene in industrial fields, water-dispersible graphene is believed to be economically and technologically useful. The generation of a large amount of oxygen functional groups (hydroxyl and carboxyl groups) on graphene led to the water dispersion of graphene; however, such over-oxidation of graphene should involve a trade-off of losing the inherent structural, electrical, and physical properties (Wang et al., 2018; Hashimoto et al., 2019). Therefore, an effective electrochemical approach is highly required to cut across such a trade-off between the high water dispersion and the over-oxidation of graphene in the electrochemical exfoliation methods.

According to the Derjaguin–Landau–Verwey–Overbeek (DLVO) theory, the aggregation of the aqueous dispersion is related to the combination of electrostatic repulsion and van der Waals attraction between particle and particle. In order to obtain a stable water dispersion of graphene, electrostatic repulsion should dominate over van der Waals attraction (Hierrezuelo et al., 2010). With the approach of introducing functional groups that can effectively offer surface charge to graphene sheets despite the small amount of functional groups, it is possible to increase the water dispersion stability without disturbing the inherent properties of graphene sheets.

In this work, we report the preparation of highly water-dispersible graphene through the mild *in situ* sulfate functionalization of graphite manipulated by controlling the reaction of persulfate (S_2_O_8_^2−^) ions to graft sulfate (R–OSO_3_^−^) groups onto the graphene sheets during the electrochemical reactions. The as-functionalized sulfate groups on graphene are believed to induce the stable water dispersion of graphene rather than the conventional oxygen functional groups such as carboxyl and hydroxyl groups; the strongly acidic sulfate groups are expected to be fully dissociated and induce strong negative charges under all relevant solution conditions, but the carboxyl or hydroxyl groups offer negative charges at a relatively higher pH value (Behrens et al., 2001). In our electrochemical regime of mild *in situ* sulfate functionalization, the highly water-dispersible graphene could be prepared with a relatively lower oxygen content of <10% compared with the inevitable over-oxidization of graphene in the conventional electrochemical exfoliation process. As a consequence, the as-derived strong negative charge of sulfate-functionalized graphene could generate the water dispersion without the generally additional separation processes of sedimentation and/or centrifugation. Here, we expect that the results regarding water-dispersible graphene produced in this study may significantly contribute to providing a high-quality graphene product to the practical industrial fields.

## Materials and Methods

### Materials

Ammonium persulfate (>98%), potassium hydroxide (>97%), and ammonium acetate (>97%) were purchased from Sigma-Aldrich. Graphite foil (99.8%) was purchased from Alfa-Aesar. All chemicals were used as received without any further purification.

### Electrochemical Exfoliation of Graphite Foil

In a typical procedure, graphite foil was used as the anode for the electrochemical exfoliation of graphite, with Pt foil being used as the cathode. As the electrolytes for the electrochemical reaction, ammonium persulfate (as an oxidant) and ammonium acetate (as pH buffer) were dissolved in deionized water using a mechanical stirrer to obtain 0.35 and 0.5 M solutions, respectively. The pH of the solution was adjusted by adding potassium hydroxide (KOH) to prevent the solution from becoming acidic (below pH 7) during the electrochemical reaction. The electrochemical exfoliation reaction was carried out by applying a constant voltage (10 V) to the graphite-foil electrode at 25°C for 1 h. The reaction product was collected by vacuum filtration and washed several times with deionized water. The resulting wet black powder was ultrasonicated in deionized water for 30 min, and a stable graphene nanosheet dispersion was obtained without any additional sedimentation and/or centrifugation processes. The dispersion stability of the graphene nanosheet could be controlled by changing the initial pH of the electrolyte.

### Measurement and Characterization

Electrochemical exfoliation was conducted by using a ZIVE MP1 electrochemical workstation (WonATech). Transmission electron microscopy (TEM) and high-resolution transmission electron microscopy (HRTEM) images of the graphene samples were acquired using a JEOL JEM-2010 microscope at an accelerating voltage of 200 kV. Atomic force microscopy (AFM) images were obtained using an XE-100 (Park Systems) instrument. X-ray photoelectron spectroscopy (XPS) results were obtained using a Kratos AXIS Ultra DLD with an Al Kα radiation source (1,486.8 eV) in an ultrahigh vacuum chamber (7 × 10^−9^ Torr). The Raman spectrum of the graphene sample was measured using a UniRAM Raman microscope (Uninanotech) at room temperature and an excitation wavelength of 532 nm. The attenuated total reflection Fourier transform infrared (ATR-FTIR) spectrum of the graphene was collected using a Nicolet iS10 FT-IR spectrometer (Thermo Scientific). The sample dispersion stability was characterized using a Leanontech Turbiscan Lab Expert instrument with a near-infrared (NIR) light source (880 nm). The zeta potential was obtained by using an ELSZ-1000 (Otsuka Electronics).

## Results and Discussion

**FIGURE 1:**
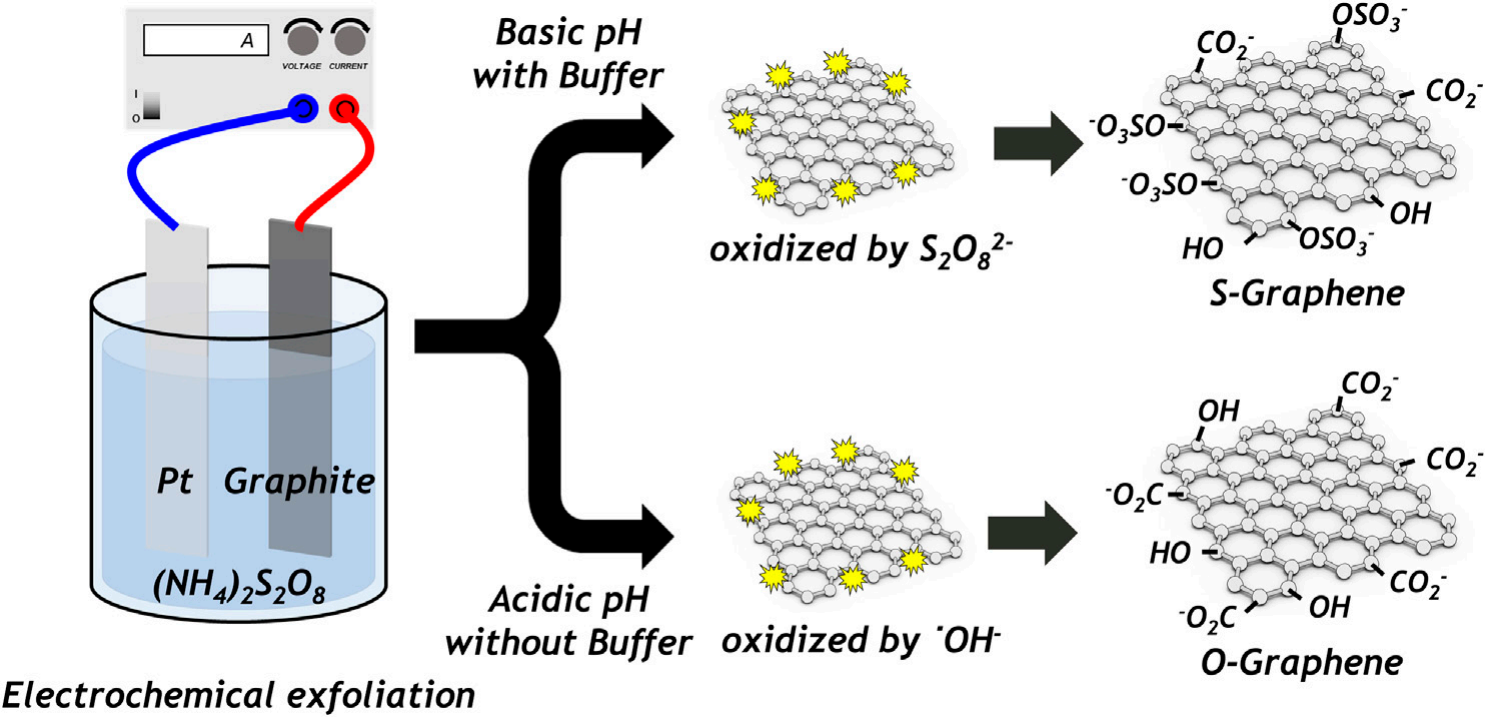
Schematic of the electrochemical exfoliation of graphite with and without pH buffer.

The persulfate anion (S_2_O_8_^2−^), a strong oxidant (*E* ^*0*^ *=* 2.01 V) (Yuan et al., 2014), undergoes chemical or thermal dissociation to become an intermediate of sulfate free radicals (SO_4_˙^−^), which can be readily converted to hydroxyl free radicals (˙OH) in aqueous solution *via* a radical interconversion reaction. In particular, under acidic conditions, the breakdown of S_2_O_8_^2−^ can be further acid-catalyzed; as a result, most of the S_2_O_8_^2−^ converts into ˙OH (Liang et al., 2007). For this reason, the electrochemical exfoliation of graphite in a persulfate electrolyte affords only oxygen functional groups (i.e., carboxyl and hydroxyl groups) grafted on graphene, which is specifically formed by ˙OH despite the use of sulfur atom–containing oxidizing reagents. To anchor sulfate functional groups onto the graphene in the electrochemical exfoliation step, it is necessary to prevent the dissociation of persulfate ions into radical species, which can be achieved by maintaining the basicity of the solution during the electrochemical reaction. Therefore, the pH of the electrolyte employed in our study was carefully kept at a value of higher than seven during the whole electrochemical reaction. By using potassium hydroxide and a pH buffer reagent, the basic condition of the electrochemical reaction (pH value > 7) was maintained until the electrochemical reactions terminate. As a result, the highly water-dispersible graphene functionalized with the sulfate groups could be obtained in our electrochemical scheme (Figure 1).

**FIGURE 2:**
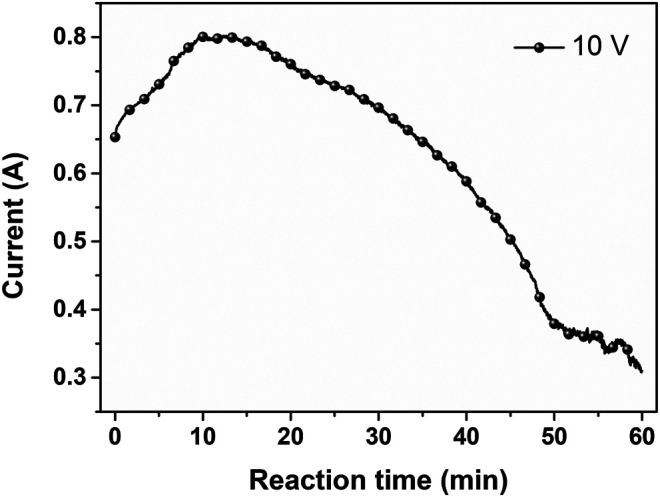
Current–time plot during the electrochemical exfoliation of graphite applying 10 V.

In the specific two-electrode system with the use of graphite foil as the anode and Pt foil as the cathode, the electrolyte solutions were first prepared by dissolving ammonium persulfate in deionized water to obtain a 0.35 M aqueous solution, and the pH value was adjusted using potassium hydroxide with ammonium acetate buffer solution (pH 11). Subsequently, a constant voltage of 10 V was applied to the graphite-foil electrode at a temperature of 25°C to prevent the thermal decomposition of persulfate ions (Liang et al., 2007). For 10 min from the beginning of applying the voltage, the current was gradually increased due to decreasing interfacial resistance between the electrode and the electrolyte by increasing the surface area of the graphite electrode, which resulted from the gas-evolved expansion of graphite. After that, as the expanded graphite electrode was eroded and exfoliated into flakes, the current was gradually decreased. The exfoliation process was almost completed with a slight change in the current at 50 min, and the reaction was terminated at 1 h (Figure 2). The as-exfoliated graphite separated in the form of a black powder was collected by vacuum filtration and repeatedly washed with deionized water to remove any residual impurities and salts. The as-collected black powder was ultrasonicated to obtain a highly concentrated aqueous graphene dispersion with a concentration of 15 mg/ml, importantly without any additional separation processes of sedimentation and/or centrifugation. It is also noted that the simple ultrasonication step could completely exfoliate the as-expanded graphite into individual graphene sheets without any dots of un-exfoliated graphite powder.

**FIGURE 3:**
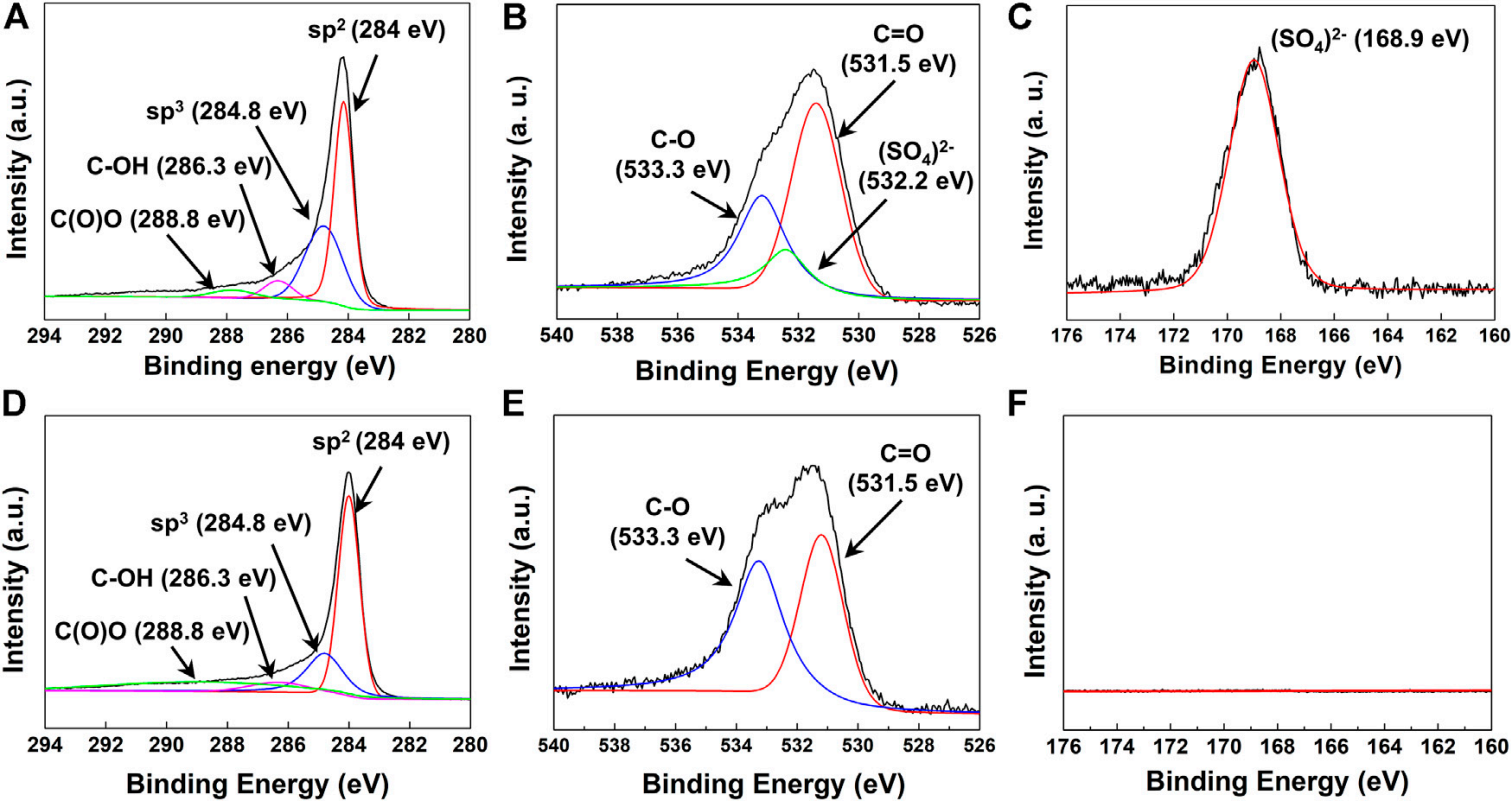
**(A)** C 1s, **(B)** O 1s, and **(C)** S 2p XPS spectra of electrochemically exfoliated graphene with pH buffer. **(D)** C 1s, **(E)** O 1s, and **(F)** S 2p spectra of electrochemically exfoliated graphene without pH buffer.

The chemical structure and bonds of water-dispersible graphene were investigated by X-ray photoelectron spectroscopy (XPS), which depicts the detailed chemical composition in the form of the peak binding energies of C 1s, O 1s, and S 2p. The sulfate-functionalized graphene could be identified as mostly consisting of sp^2^-type carbon as per the peak at 284.0 eV with a small amount of sp^3^-type, hydroxyl-type, and carboxyl-type carbon, corresponding to peaks at 284.8, 286.3, and 288.8 eV, respectively (Figure 3A) (Parvez et al., 2014). As the peak of the C–S bond (285.2 eV) (Ye et al., 2015) could not be observed, we notice that the sulfonate group (R–SO_3_^−^) has not been grafted on the surface of graphene. Instead, we could observe the chemical bonds between carbon and oxygen (533.3 eV for C–O and 531.5 eV for C=O) and between sulfur and oxygen (SO_4_^2−^, 532.2 eV) (Figure 3B) (Kwan et al., 2015; Singh et al., 2018) from the O 1s peaks. Moreover, the peak corresponding to the sulfate bond (SO_4_^2−^, 168.9 eV) is found in the S 2p spectra, without other peaks corresponding to the sulfonate (SO_3_^2−^, 167.5 eV) bond (Figure 3C) (Massonnet et al., 2015). Based on the results of XPS analysis, the water-dispersible graphene nanosheets are signified to be sulfate-functionalized where sulfur atom–containing functional groups were introduced in the form of sulfate (R–OSO_3_^−^) instead of sulfonate (R–SO_3_^−^). Furthermore, the FT-IR spectrum of graphene collected in the ATR mode also revealed that water-dispersible graphene has various types of functional groups in the structure (Supplementary Figure S1). The distinctive peak was observed at 1,170 cm^−1^ and 1,060 cm^−1^, corresponding to the stretching vibration of the sulfate group (Guo et al., 2019), while the vibrational bands at 3,400 cm^−1^ and 1720 cm^−1^ correlate with hydroxyl and carboxyl, respectively (Aunkor et al., 2016).

The key role of the mild *in situ* sulfate functionalization of graphene employed in our study for the production of water-dispersible graphene could be confirmed in comparison to the hydrophobic graphene produced without maintaining the basic pH of the electrolyte during the electrochemical reaction. Different from the specific electrochemical reaction of water-dispersible graphene, we conducted the same electrochemical reaction without using the buffer reagent. In detail, we carried out the electrochemical reaction in the solution with the concentration of 0.35 M, adjusting the pH of the electrolyte to 11, applying the constant voltage of 10 V at a temperature of 25°C, continuing the reaction for 1 h, and only without using the buffer reagent of ammonium acetate. Without using the buffer reagent, the pH value of the electrolyte changes from 6.5–7 to 2 during the electrochemical reaction because the persulfate ions dissociate into sulfuric acid as the reaction proceeds (Liang et al., 2007). As a result, the comparison reaction condition yielded only the hydrophobic graphene sheets that could only be dispersed in organic solvents (e.g., dimethylformamide and N-methylpyrrolidone) instead of water. From the hydrophobic graphene prepared without the buffer solution, the peak C 1s positions are similar to those of the sulfated graphene sheets at 284.0, 284.8, 286.3, and 288.8 eV, corresponding to sp^2^-, sp^3^-, hydroxyl-, and carboxyl-type carbon, respectively (Figure 3D). The peaks at 533.3 and 531.5 eV correspond to C–O and C=O binding about O 1s, respectively (Figure 3E). However, no peaks are observed at 532.2 eV in the O 1s spectra and at 168.9 eV in the S 2p spectra, which, importantly, indicates the absence of sulfur-containing functional groups on the graphene sheet without the buffer solution (Figure 3F).

To confirm the effect of pH on the sulfate functionality and surface charges of graphene sheets generated during the electrochemical exfoliation process, we used XPS to analyze the sulfate-functionalized graphene obtained by changing the initial pH value to determine the degree of sulfation, according to the atomic concentration of carbon and sulfur from the peak areas of C 1s and S 2p, respectively. The zeta-potential analyzer was used to measure the surface zeta potential (ζ) in neutral deionized water (Table 1). It is noticed in Table 1 that as the initial pH value increases from 8 to 12, the carbon atomic concentration decreases from 90.17 to 89.12% and the sulfur atomic concentration increases from 0.72 to 1.60%, with the relatively low oxygen contents of <10%, respectively. In other words, as the initial pH value of the solution in the electrochemical exfoliation reaction increases, the S/C ratio increases from 0.008 to 0.018, indicating the stronger sulfate functionalization of graphene under the strong basic conditions. Moreover, the surface zeta potential increases from −25.40 mV to −34.68 mV in accordance with the increase in the initial pH value. Based on the relationship between the atomic ratios and the initial pH values of electrolytes, we may draw a conclusion that the strong sulfate functionalization of graphene corresponds to the graphene sheet with the higher surface zeta potential.

**TABLE 1:** Elemental percentage, atomic ratio, and surface zeta potential of graphene sheets with different initial pH values during electrochemical exfoliation.

	pH 8	pH 9	pH 10	pH 11	pH 12
C (%)	90.17	89.83	89.28	88.38	89.12
O (%)	9.11	9.27	9.56	10.21	9.28
S (%)	0.72	0.90	1.16	1.41	1.60
S/C	0.008	0.010	0.013	0.016	0.018
ζ (mV)	−25.40	−28.73	−30.91	−33.34	−34.68

**FIGURE 4:**
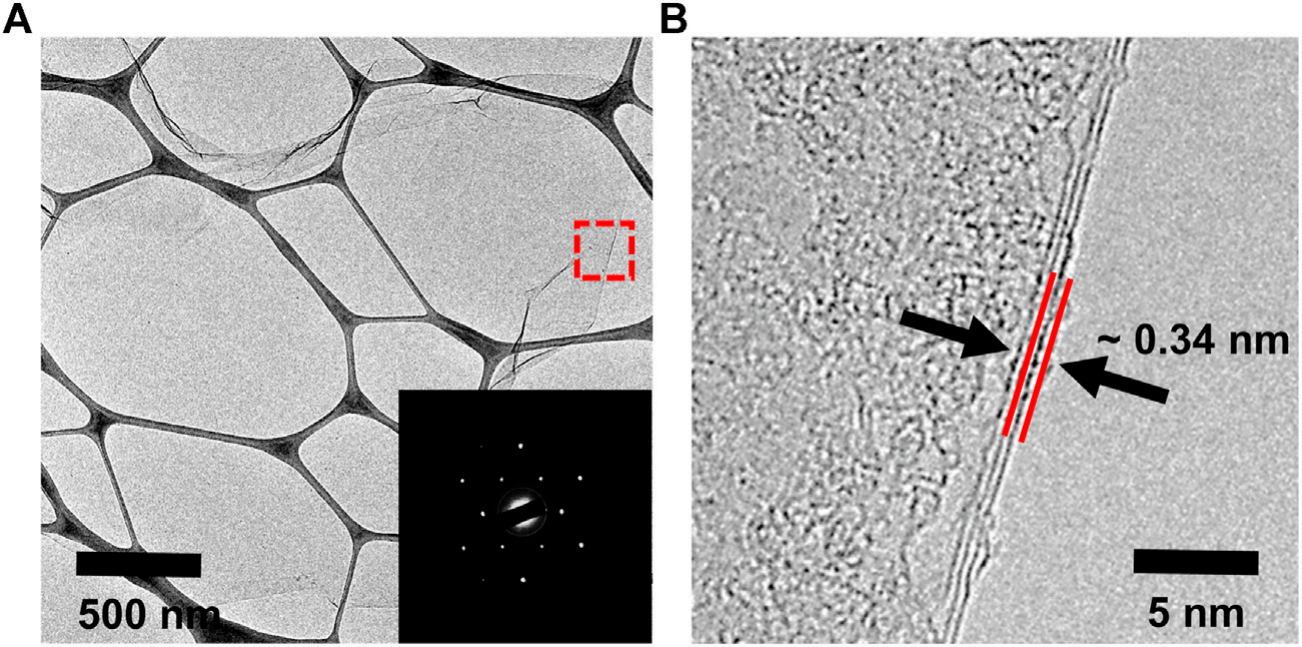
**(A)** TEM image and SAED pattern (inset) and **(B)** HRTEM image of the graphene sheet prepared with pH buffer at an initial pH value of 11.

**FIGURE 5:**
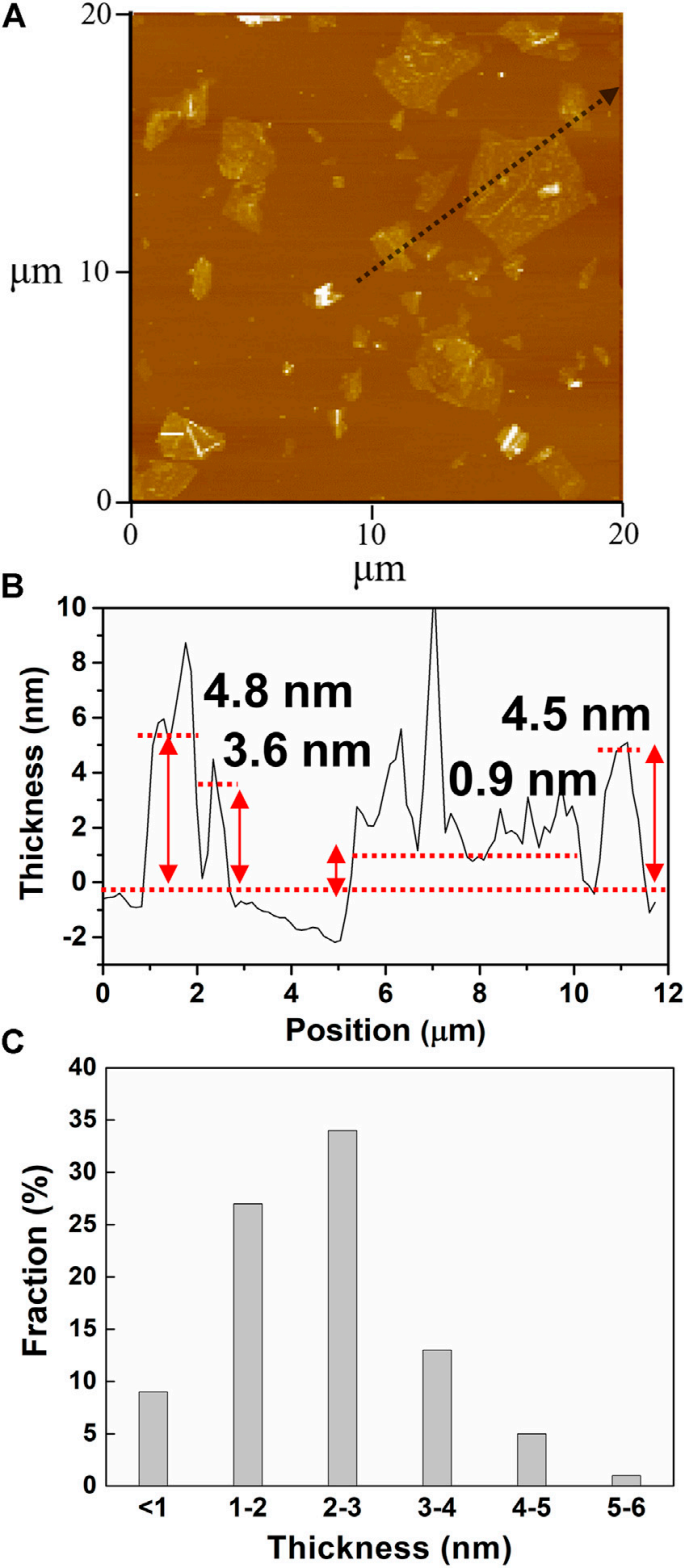
**(A)** AFM image of the graphene sheets prepared with pH buffer at an initial pH value of 11. **(B)** Height profile of a line scan about the black arrow in Figure 5A. **(C)** Histogram of thickness measurements of 100 graphene sheets.

The microstructures of water-dispersible graphene were observed by high-resolution transmission electron microscopy (HRTEM), as shown in Figure 4. The water-dispersible graphene sheet is few-layered graphene with a lateral size of several micrometers. In addition, the selected area electron diffraction (SAED) pattern represents the high crystallinity in the basal plane of the graphene sheet, where carbon atoms array in a hexagonal lattice structure (the inset), as generally observed in the electrochemically exfoliated graphene. Such high crystallinity of water-dispersible graphene is further confirmed by observing hexagonal SAED patterns at various points on the basal plane of a single graphene sheet (Supplementary Figure S2). The typical atomic force microscopy (AFM) images shown in Figure 5 exhibit the graphene sheets with the lateral sizes ranging approximately from 1 to 5 µm (Figures 5A,B) and the thickness measurement results for 100 graphene sheets, with the average thickness of graphene sheets being <5 nm (Figure 5C).

**FIGURE 6:**
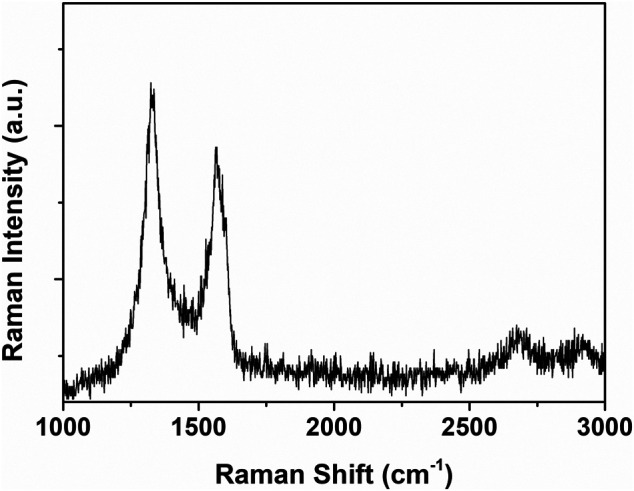
Raman spectrum of the graphene sheets prepared with pH buffer at an initial pH value of 11.

The compositional characteristics of water-dispersible graphene were investigated by Raman spectroscopy, which is an important tool to characterize graphene materials. The Raman spectrum provides information about the surface disorder and the graphitic composition of graphene materials together with the number of the graphene layers, which was expressed in the D band, the G band, and a weak 2D band, respectively (Kudin et al., 2008). The water-dispersible graphene showed the D band at 1,340 cm^−1^, the G band at 1,570 cm^−1^, and the weak 2D band at 2,680 cm^−1^ (Figure 6). The intensity ratio of bands D and G (I_D_/I_G_) is used to investigate the degree of disorders in graphene. Contrary to the TEM analysis result, it shows a high I_D_/I_G_ value of 1.03, similar to rGO (Aunkor et al., 2016), which is a result of numerous edge defects originating from nanocrystalline domains of water-dispersible graphene. From the Knights empirical equation expressed by L_a_ = 4.35(I_D_/I_G_)^−1^, the size of the sp^2^ carbon domains (L_a_) in the water-dispersible graphene was found to be 4.23 nm (Tai et al., 2010).

**FIGURE 7:**
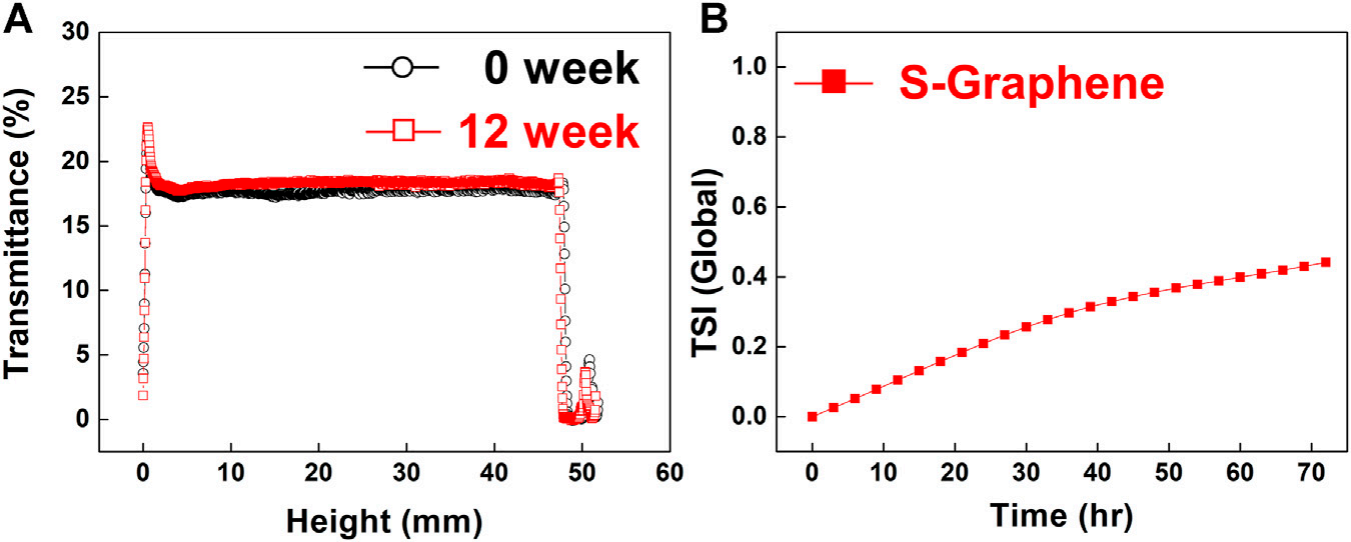
**(A)** Transmittance of aqueous dispersion of water-dispersible graphene sheets as a function of the solution height at 0 and 12 weeks. **(B)** TSI of the dispersion over time.

The dispersion stability of water-dispersible graphene in aqueous solutions (0.01 wt%) over 3 days was quantitatively analyzed using a multi-light scattering technique (Figure 7A), in which the dispersion stability is confirmed by studying the variations in transmittance across the entire height of the sample solution (Terayama et al., 2003). As recognized from the results, the highly stable dispersion presents the constant transmittance regardless of the solution height, and there is very little change in the transmittance over time. From Figure 7A, it is noted that the transmittance of the water dispersion of graphene ranges from 17.5 to 18.5% across the entire height of the sample; moreover, the transmittance remains in the range of 18.1–19.2% even after 12 weeks. A slight increase in the transmittance over time is also observed owing to the minimized flocculation and/or coalescence of graphene. To better understand the water dispersion stability of graphene, we numerically calculated the turbiscan stability index (TSI) from the mean kinetics value of the transmittance over time. The value of the TSI was found to slightly increase from 0 to 0.4%, indicating the high water dispersion stability of graphene (Figure 7B).

## Conclusion

In summary, we report the preparation of highly water-dispersible graphene using electrochemical exfoliation of graphite *via* mild *in situ* sulfate functionalization by controlling the reaction of S_2_O_8_^2−^ ions. Under the basic pH conditions (>pH 7) during the electrochemical exfoliation of graphite, the sulfate functionalization of graphene could be effectively induced, and as a result, the water-dispersible graphene could be prepared with high crystallinity and a relatively low oxygen content of <10% compared with graphene obtained from conventional electrochemical exfoliation. The aqueous dispersion of sulfated graphene was highly stable over a long time without any additional separation processes of sedimentation and/or centrifugation, exhibiting constant transmittance regardless of the solution height and very little change in the transmittance and the TSI. This result indicates that the sulfate functional group attached to the graphene sheet effectively stabilizes the dispersion from electrostatic repulsive forces by increasing the surface charge even when only a small amount of sulfate functional groups is present. More importantly, our study shows that a comprehensive understanding of the persulfate reaction can provide a tool to introduce sulfate functional groups in various materials and increase dispersion stability without inhibiting their original characteristics.

## Data Availability

The original contributions presented in the study are included in the article/Supplementary Material; further inquiries can be directed to the corresponding authors.
