# Supplementary material for: Highly Water-Dispersible Graphene Nanosheets From Electrochemical Exfoliation of Graphite
Source: Front Chem. 2021 Jul 21;9:699231. doi: 10.3389/fchem.2021.699231 (PMC8335538; doi:10.3389/fchem.2021.699231)
Supplement: Supplementary file 1 [file DataSheet1.docx]

Supplementary Material

Highly Water-Dispersible Graphene Nanosheets from Electrochemical Exfoliation of Graphite

Si-Woo Park^1, 2, †^, Byungkwon Jang^1, †^, Han Kim^1^, Jimin Lee^1^, Ji Young Park^1^, Sung-Oong Kang^2, *^, Yong-Ho Choa^1, *^

^1^Department of Material Science & Engineering, Hanyang University, Ansan, Korea

^2^MExplorer Co., Ltd., Ansan, Korea

^†^These authors have contributed equally to this work and share first authorship

*** Correspondence:**Corresponding Author
Sung-Oong Kang; Yong-Ho Choa
kso3710@gmail.com
choa15@hanyang.ac.kr

**Supplementary Data**





Figure S1. FT-IR spectrum of of the electrochemically exfoliated graphene with pH buffer.

**
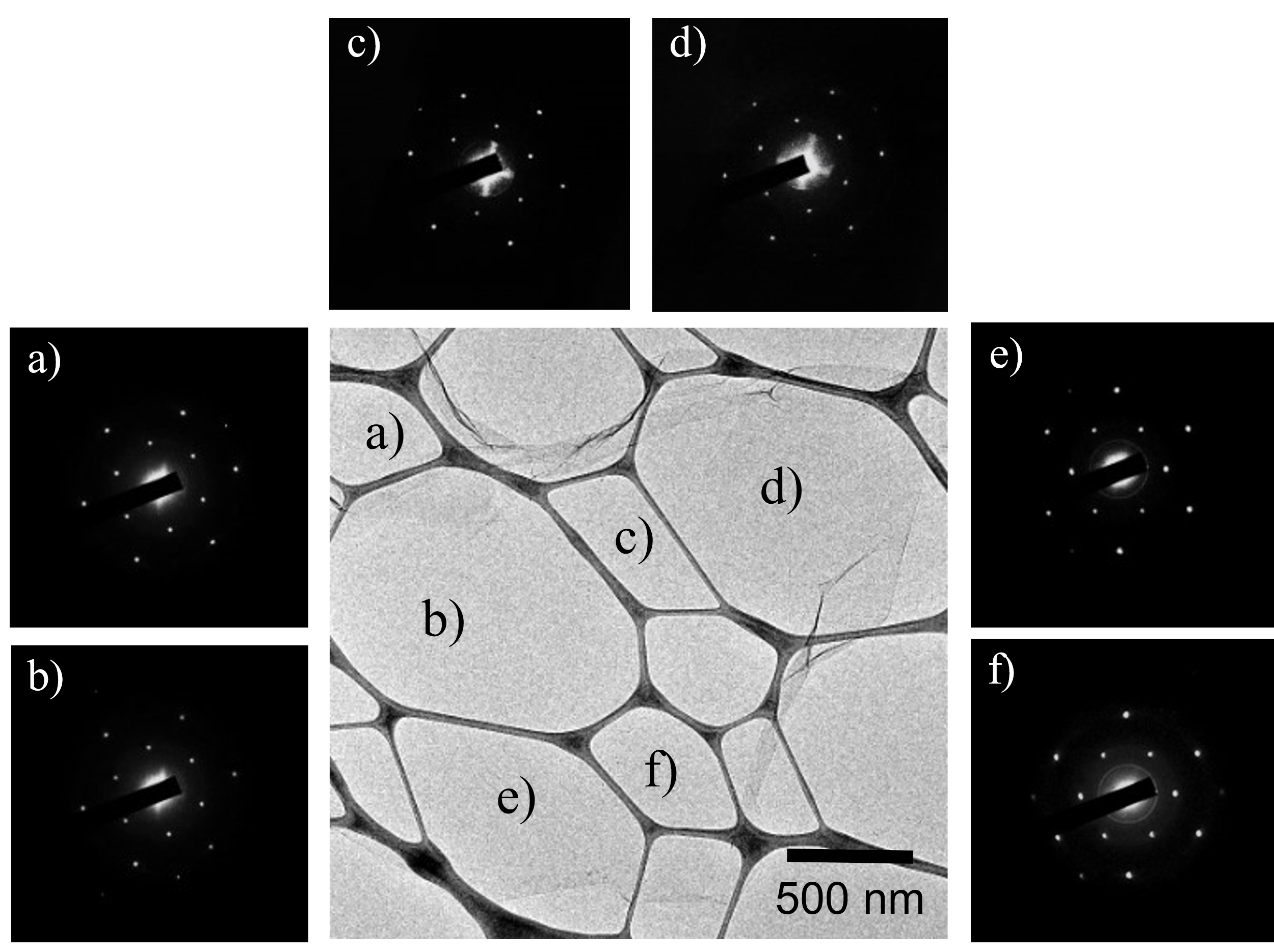
**

**Figure S2.** TEM image and SAED patterns corresponding to each part in the graphene sheet.


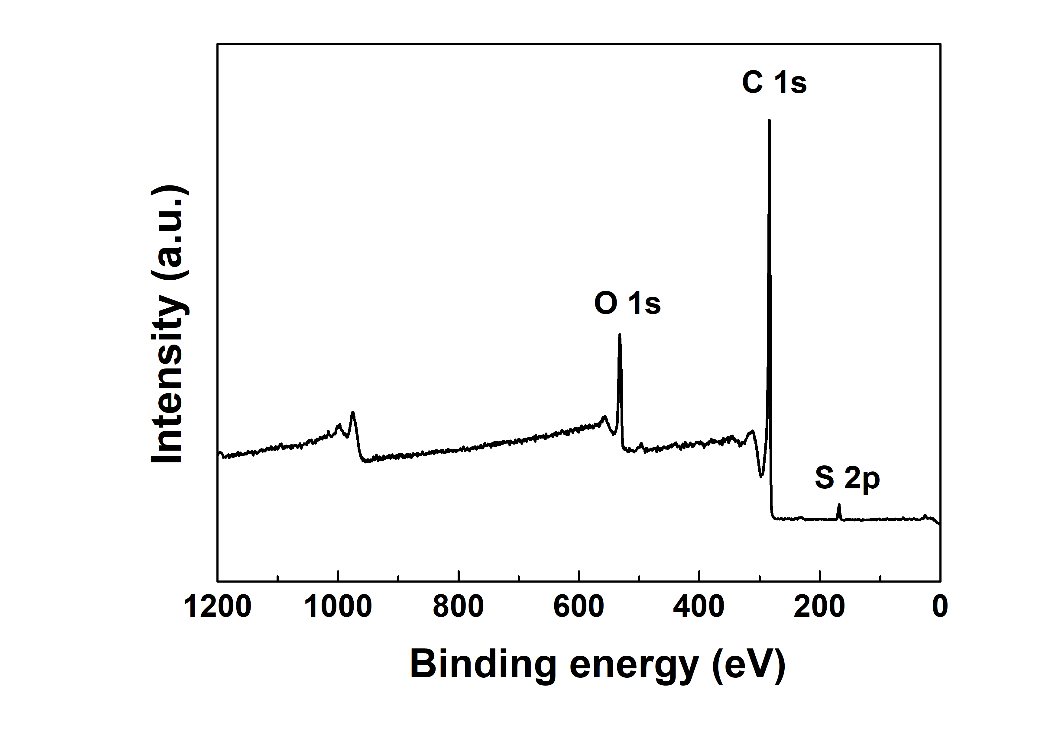


Figure S3. Full scan XPS spectra of the electrochemically exfoliated graphene with pH buffer.


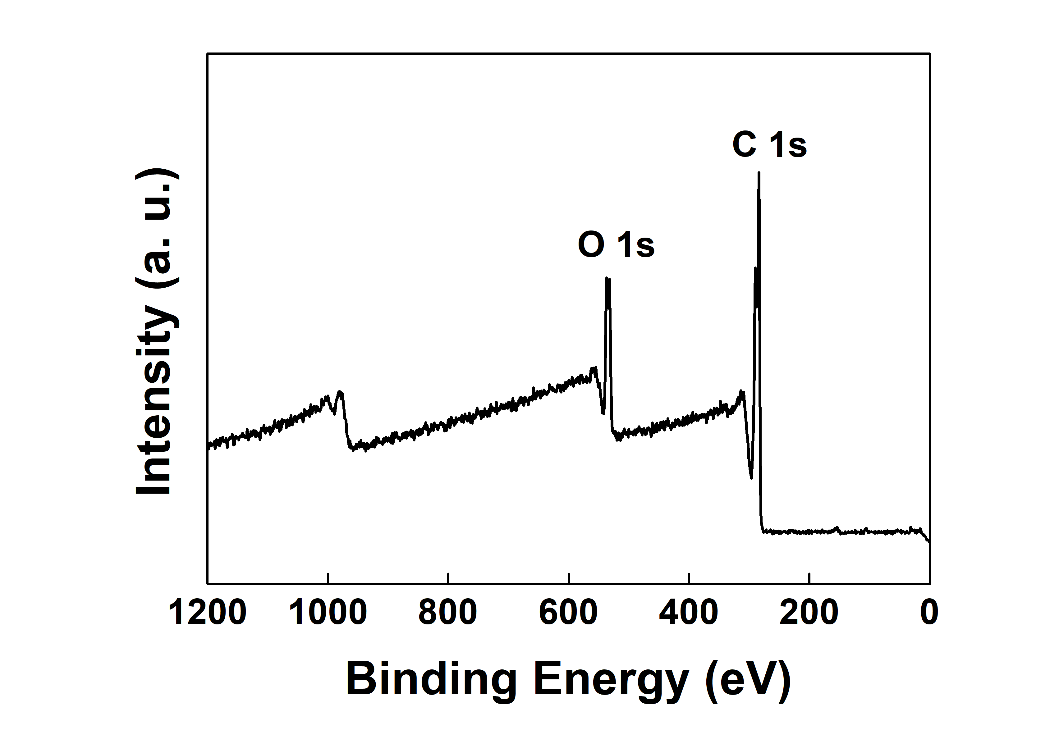


Figure S4. Full scan XPS spectra of the electrochemically exfoliated graphene without pH buffer.
